# Cognitive reserve and lifestyle: moving towards preclinical Alzheimer’s disease

**DOI:** 10.3389/fnagi.2015.00134

**Published:** 2015-08-10

**Authors:** Eider M. Arenaza-Urquijo, Miranka Wirth, Gaël Chételat

**Affiliations:** ^1^INSERM, U1077Caen, France; ^2^Université de Caen Basse-Normandie, UMR-S1077Caen, France; ^3^Ecole Pratique des Hautes Etudes, UMR-S1077Caen, France; ^4^CHU de Caen, U1077Caen, France

**Keywords:** cognitive reserve, brain reserve, neuroimaging, Alzheimer’s disease, amyloid, preclinical Alzheimer’s disease, compensation, neuroprotection

## Abstract

The large majority of neuroimaging studies in Alzheimer’s disease (AD) patients have supported the idea that lifestyle factors may protect against the clinical manifestations of AD rather than influence AD neuropathological processes (the cognitive reserve hypothesis). This evidence argues in favor of the hypothesis that lifestyle factors act as moderators between AD pathology and cognition, i.e., through indirect compensatory mechanisms. In this review, we identify emerging evidence in cognitively normal older adults that relate lifestyle factors to established AD neuroimaging biomarkers. While some of these investigations are in agreement with the compensatory view of cognitive reserve, other studies have revealed new clues on the neural mechanisms underlying beneficial effects of lifestyle factors on the brain. Specifically, they provide novel evidence suggesting direct effects of lifestyle factors on AD neuropathological processes. We propose a tentative theoretical model where lifestyle factors may act via direct neuroprotective and/or indirect compensatory mechanisms. Importantly, we suggest that neuroprotective mechanisms may have a major role during early stages and compensatory mechanisms in later stages of the disease. In the absence of an effective treatment for AD and considering the potential of lifestyle factors in AD prevention, understanding the neural mechanisms underlying lifestyle effects on the brain seems crucial. We hope to provide an integrative view that may help to better understand the complex effects of lifestyle factors on AD neuropathological processes, starting from the preclinical stage.

## Introduction

In the absence of effective treatments for Alzheimer’s disease (AD), new therapeutical approaches that may assist in preventing and/or delaying the onset of AD appear essential. Lifestyle factors such as cognitive, social and/or physical activities have a great potential for AD prevention (Barnes and Yaffe, 2011; Erickson et al., 2012). Therefore, understanding the biological mechanisms underlying lifestyle effects on the brain seems crucial.

During the last decades, several theories have provided a conceptual framework to study the effects of lifestyle factors on the brain. In the present review, we are interested in cognitive and brain reserve theories (Stern, 2002; Satz et al., 2011), with special interest in cognitive reserve. Cognitive reserve is a pivotal concept that has driven several neuroimaging investigations, and has found support in the vast majority of these investigations in AD. More recently, notably due to the development of β-amyloid (Aβ) ligands and the definition of preclinical AD (Sperling et al., 2011), new evidence has arisen from studies in cognitively normal older adults. These studies relate lifestyle factors to AD neuropathological processes by means of AD neuroimaging biomarkers (i.e., Aβ deposition, atrophy and hypometabolism). While cognitive reserve argues in favor of lifestyle factors as moderators between AD pathology and cognition, implying that lifestyle effect would be independent of AD pathology (Stern, 2002), we will identify emerging neuroimaging evidence in cognitively normal older adults showing a direct effect of lifestyle factors on AD neuropathological processes.

The review is organized as follows. First, we provide a brief overview of cognitive and brain reserve theories. Then, we focus on evidence that relate AD neuroimaging biomarkers (i.e., Aβ deposition, hypometabolism and atrophy) to lifestyle factors, first in AD patients and then in cognitively normal older adults. Evidence focused on the relationship between lifestyle factors and cognitive performances will not be included in the present review. Moreover, although cognitive and brain reserve theories have also been investigated in other neurodegenerative diseases, such as frontotemporal dementia, Parkinson disease and Huntington’s disease (Borroni et al., 2012), this review only elaborates on the concept of reserve in the framework of neuroimaging research in AD. In the last section of the review, we discuss the potential mechanisms underlying the effects of lifestyle factors, i.e., neuroprotection and compensation, also taking into account evidence from other fields such as research in animals and intervention studies. We emphasize recent investigations in preclinical AD that have provided new insights into the field. Overall, we hope to provide an integrative view that may represent a first step towards a broader theoretical model that considers both indirect and direct effects of lifestyle factors on AD neuropathological processes, extending from cognitively normal older adults to AD patients.

## Brief Definition of Cognitive and Brain Reserve and Current Approach

In the field of aging and dementia, the concept of “reserve” stems from the repeated observation that there is a discrepancy between the degree of neuropathological changes associated with aging and AD and their clinical expression (for example, Katzman et al., 1989). Thus, “reserve” refers to the hypothetical capacity of the brain to tolerate more aging or pathological effects, thereby reducing the impact on clinical symptoms (Stern, 2002).

There have been different attempts to conceptualize “reserve” including passive (Katzman, 1993; Satz et al., 1993; Mortimer, 1997) and active models. The passive model revolves around brain reserve concept and refers to quantitative characteristics that may help to tolerate adverse effects of neuropathology, such as brain size (Katzman, 1993) or neuronal count (Mortimer et al., 1981). According to the passive model, there is a threshold at which clinical deficits will become apparent and those subjects with more brain reserve (e.g., bigger brains) require more pathology to reach that threshold (Satz et al., 1993). The active model revolves around cognitive reserve concept and refers to how flexibly and efficiently an individual can make use of available brain reserve (Stern, 2002). Cognitive reserve implies variability at the level of brain networks (Stern, 2009), i.e., interindividual differences in the ability to use brain networks in an effective way. This includes preexisting differences in neural preprocessing (neural reserve), and alterations in neural processing that may take place in order to cope with increasing brain pathology (neural compensation; Stern, 2009). Thus, while brain reserve emphasizes the anatomical potential or brain structure, cognitive reserve emphasizes brain function.

It needs to be noted that reserve is a hypothetical construct and thus, direct measures of reserve are not available (Jones et al., 2011). Therefore, surrogate or proxy measures are used to approach reserve. Proposed surrogate or proxy measures of brain reserve include measures of neuronal number, dendritic arborization or synapse density (Stern, 2002). Measures such as brain volumes (Solé-Padullés et al., 2009) intracranial volume (ICV, as a proxy for maximal brain volume) or head circumference (for example, Mortimer et al., 2005) have also been used in the literature. In addition, based on solid epidemiological evidence (see for example, Meng and D’Arcy, 2012), education (Stern et al., 1992) and IQ (Alexander et al., 1997) are also considered as standard proxies of cognitive reserve. This has been extended to include other proxies such as occupation (Stern et al., 1994; Richards and Sacker, 2003; Staff et al., 2004), engagement in leisure and cognitive activities (Scarmeas et al., 2001, 2003a; Wilson et al., 2002), social networks (Fratiglioni et al., 2000; Bennett et al., 2006), dietary habits (Scarmeas et al., 2006) and more generally a mental stimulating lifestyle clustered around the concept of cognitive lifestyle (Valenzuela and Sachdev, 2006). Performance-based measures (such as vocabulary or reading tests) have also been used as they are thought to show little change with age and remain relatively preserved in the early stages of dementia (for example, Park et al., 2002; Wisdom et al., 2012). Finally, factors reflecting the variance between two or more of these variables (Scarmeas et al., 2004; Bartrés-Faz et al., 2009; Solé-Padullés et al., 2009; Bosch et al., 2010) have also been used. The present review considers proxies or surrogates of reserve as reflecting lifestyle involvement in cognitive (including educational attainment and occupation), physical and social activities. These proxies will be referred to as lifestyle factors or positive lifestyle factors throughout the manuscript to avoid confusion. We recognize the effect of these variables not only in relation to the concept of cognitive reserve, but also above this concept. Indeed we acknowledge the possibility that these factors may impact on the manifestation of AD not only through cognitive reserve mechanisms but also by exerting their impact on the neuropathological processes of AD. Finally, although epidemiological data suggested other modulating factors that may alter the clinical expression of the disease (i.e., vascular risk factors such as hypertension, hypercholesterolemia and diabetes) they were not within the scope of the present review.

The present review is based on the assumption that cognitive and brain reserve theories may provide independent as well as interactive contributions, because of different reasons. First, functional differences in cognitive processing (cognitive reserve) might be sustained by an anatomical substrate (brain reserve; Stern, 2009). Furthermore, brain reserve proxies (which are a static measure from a strict theoretical point of view) might be modifiable through lifestyle variables, as shown for example with brain volumes in neuroimaging and interventional studies (see for example, Erickson et al., 2011). Thus, lifestyle variables may influence both brain structure (brain reserve) and function (cognitive reserve; Bartrés-Faz et al., 2009; Arenaza-Urquijo et al., 2013a). Finally, evidence from studies considering a life course approach to the effects of lifestyle suggests that a genetically endowed advantage may lead, for example, to larger brains (brain reserve) and may then influence individual’s choices such as education, occupation, intellectual and physical activities (Staff et al., 2012), which may in turn impact cognitive reserve. Thus, the differentiation between cognitive and brain reserve seems to be strictly theoretical and somehow artificial. However, different methodological approaches have been used to study these two concepts. In the present review, we assume that lifestyle factors may influence both brain structure and function. Studies that do not assess the relationship between lifestyle factors and brain measures but the characteristics that differentiate a control group from a case group (for example, cognitively normal Aβ positive as compared to Aβ negative subjects) would be considered under the brain reserve model.

## Cognitive Reserve, Lifestyle and Alzheimer’s Disease Neuroimaging Markers: from Alzheimer’s Disease Dementia to Preclinical Stages

### Summary of Previous Findings in Alzheimer’s Disease Patients

**Figure 1:**
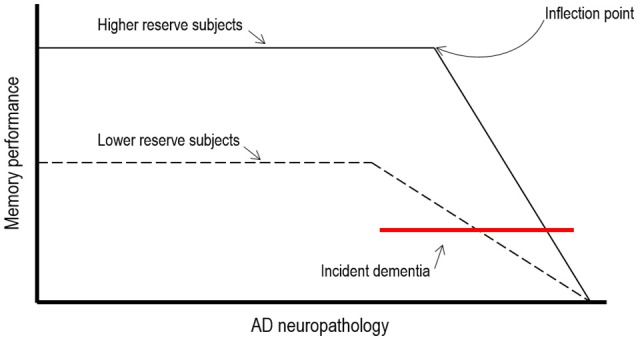
**Theoretical illustration of how cognitive reserve may mediate the relationships between AD neuropathology and cognitive function (adapted from Stern, 2009)**.

In AD patients cognitive reserve theory predicts that the clinical manifestation of advancing AD pathology would be delayed in patients with higher exposure to cognitive, social and physical activities. This implies first, that neuropathological effects of AD would need to be more severe for the clinical symptoms of dementia to become evident and second, that more resources might be available in these individuals which may allow them to maintain cognitive function (see Figure 1).

A common approach in neuroimaging investigations consists in assessing the relationships between: (i) lifestyle factor(s); (ii) a marker of AD pathology (such as hypometabolism, atrophy or Aβ deposition); and (iii) cognition/clinical severity. Very often, this approach turned into a model where the relationships between lifestyle variables and neuroimaging measures have been assessed controlling for clinical severity. Consequently, given comparable clinical severity, positive lifestyle variables are expected to correlate with greater AD pathological changes pathology, thus indicating a higher tolerance. This first approach has provided strong evidence that neuroimaging biomarkers are more affected at a given level of cognitive performance in patients with higher exposure to positive lifestyle factors, when compared to patients with lower exposure to these factors. This has been shown for neuroimaging markers of perfusion in mild to moderate AD patients (Stern et al., 1992, 1994; Liao et al., 2005), glucose metabolism in AD (Alexander et al., 1997; Kemppainen et al., 2008) and mild cognitive impairment (MCI) converters (Garibotto et al., 2008), atrophy in MCI and AD (Kidron et al., 1997; Querbes et al., 2009; Solé-Padullés et al., 2009; Seo et al., 2011) and Aβ deposition in AD patients (Kemppainen et al., 2008). When the topography of the relationships has been investigated (for example using voxel-wise approaches), studies generally point to brain areas known to be vulnerable in AD, such as temporal and parietal areas (including medial areas such as the hippocampus and the precuneus) and the ventrolateral frontal cortex (for Aβ deposition). However, metabolism/ perfusion changes in other brain areas (not specifically sensitive to AD) have also been highlighted in previous studies, such as frontal (Stern et al., 1994; Alexander et al., 1997; Scarmeas et al., 2003b) or occipital (Scarmeas et al., 2003b) regions.

Another frequently used approach consists in assessing the relationships between neuroimaging markers and cognitive performances and then identifying the interactions between lifestyle factors and AD neuropathology on cognition. In other words, these investigations study whether lifestyle variables modulate the relationship between AD pathology and cognitive function. Roe et al. (2007) for example showed that Aβ deposition (measured using PIB uptake) interacted with years of education in predicting cognitive function. Performance on cognitive measures increased with increasing education for participants with elevated Aβ deposition but education was not related to global cognitive functioning scores among participants with low Aβ deposition. This suggests that individuals with more years of education were able to maintain cognitive function in the presence of cortical Aβ. Independent effects of lifestyle factors and AD pathology on cognition have been found in subsequent studies by Vemuri et al. (2012) in a pooled group of MCI and AD patients and using both structural abnormalities in AD regions and cerebrospinal (CSF) Aβ_1–42_ levels (see also “Preclinical Alzheimer’s Disease: Lifestyle Effects on AD Neuroimaging Biomarkers” Section) and a cognitive score compelling several domains (language, memory, executive, and visual); and by Rentz et al. (2010) in a pooled sample of healthy older adults and AD patients using Aβ deposition measured with PIB-PET and including a wide range of cognitive domains as well as a challenging memory test (see also “Preclinical Alzheimer’s Disease: Lifestyle Effects on AD Neuroimaging Biomarkers” Section).

Overall, studies in AD patients (some of them also including MCI patients) showed greater AD pathology in patients with higher exposure to positive lifestyle factors compared to patients with lower exposure (at the same cognitive level), consistent with the idea that reserve increases the tolerace to AD pathology. However, it needs to be noted that the reverse findings (more preserved brains in AD patients with higher exposure to positive lifestyle factors) have also been reported in some studies (Lo et al., 2013; Shpanskaya et al., 2014) as discussed below.

### Preclinical Alzheimer’s Disease: Lifestyle Effects on AD Neuroimaging Biomarkers

In the last years, notably with the emergence of Aβ imaging and the establishment of preclinical AD criteria (Sperling et al., 2011), new findings have emerged in cognitively normal older individuals. These studies included: (i) samples of cognitively normal older adults with neuroimaging biomarker information (and CSF markers in some of them); or (ii) older adults considered to be at higher risk for AD (that may thus in part represent the preclinical AD stage), such as older adults showing pathological levels of Aβ deposition or carrying the ε4 allele of the Apolipoprotein (APOE) gene (the highest known genetic risk factor for sporadic AD).

While some of these investigations are in agreement with the notion that lifestyle factors may have a modulatory effect on the brain, other studies provide new clues on the neural mechanisms underlying the effects of lifestyle factors on the brain and thus on the potential mechanisms underlying cognitive and brain reserve. These studies: (i) propose that certain anatomical or functional characteristics (for example, greater temporal volume or metabolism) may delay the deleterious effect of Aβ; and interestingly; (ii) provide novel evidence showing a direct effect of lifestyle variables on AD neuropathological processes, for example, by slowing Aβ deposition.

#### Lifestyle Effects in Cognitively Normal Individuals at Higher Risk for AD

**Figure 2:**
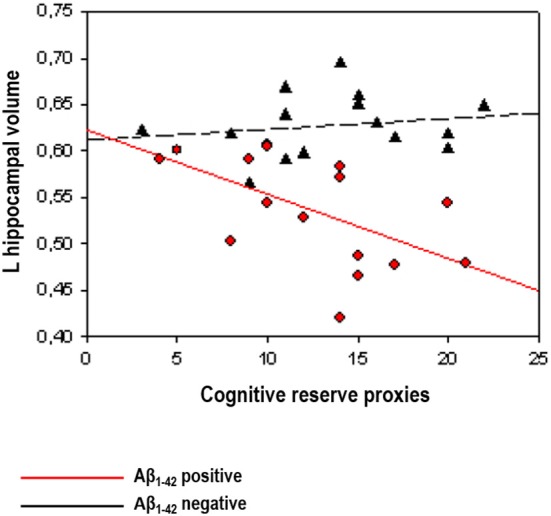
**Illustration of the interactions between Aβ status (positive vs. negative) and cognitive reserve proxies on hippocampal volume reprinted from Arenaza-Urquijo et al. (2013b; with permission from IOS Press)**. The scatterplot shows an inverse relationships between cognitive reserve markers and AD neuroimaging biomarkers restricted to cognitively normal Aβ positive subjects (in red).

A first series of studies investigated the relationships between lifestyle and neuroimaging markers controlling for cognitive performance (see above for justification), in samples of cognitively normal Aβ positive subjects classified by means of CSF Aβ_1–42_ levels. In a sample from the prospective Alzheimer’s Disease Neuroimaging Initiative (ADNI) study, Ewers et al. (2013) found that higher education was associated with lower FDG-PET glucose metabolism in Aβ positive, but not in Aβ negative subjects. In the same vein, Arenaza-Urquijo et al. (2013b), using a comprehensive proxy of cognitive reserve, reported that relatively to Aβ negative, Aβ positive subjects with higher scores on this variable presented greater atrophy in the hippocampus and cortical thinning in the supramarginal gyrus. In agreement with previous evidence in AD, authors claimed that in asymptomatic individuals with abnormal levels of Aβ (which may reflect the preclinical stage of AD) higher exposure to positive lifestyle variables compensates FDG-PET hypometabolism or gray matter atrophy/cortical thinning to maintain cognitive performance (see Figure 2). Using a different approach, Rentz et al. (2010) were able to show that a cognitive reserve factor (education and reading test) could moderate the relationships between Aβ deposition and cognitive performances in a sample including cognitively normal and mild AD patients (see also "Preclinical Alzheimer’s Disease: Lifestyle Effects on AD Neuroimaging Biomarkers” Section). In the subsample of cognitively normal older adults, they showed that Aβ deposition in the precuneus was associated with reduced memory performance and this relationship was weaker in subjects with higher cognitive reserve proxies, suggesting that cognitive reserve may be protective against Aβ-related cognitive impairment.

The studies by Chételat et al. (2010) and Johnson et al. (2014) provide complementary information. Rather than studying the relationships between lifestyle variables and brain measures, these studies highlighted the differences between cognitively normal older adults with high (Aβ-positive) vs. low (Aβ-negative) Aβ deposition. Chételat et al. (2010) showed that asymptomatic Aβ-positive had larger temporal (including hippocampal) gray matter volume than Aβ-negative individuals, which was associated with better episodic memory performance. In the same vein, Johnson et al. (2014) reported increased metabolism in the superior temporal lobe, and increased gray matter in the lateral parietal lobe, in Aβ-positive compared to Aβ-negative subjects. In accordance with the brain reserve hypothesis, we could suggest that the deleterious effects of Aβ deposition on cognition might be delayed in individuals with higher gray matter volumes or higher glucose metabolism. Although these mechanisms may be somehow passive, neural compensation related to more detailed memory encoding in older adults with Aβ deposition has also been reported (Elman et al., 2014). Thus, cognitively normal older adults with Aβ deposition may recruit extra neural resources, including increases in brain activation in the parietal and occipital cortex that may allow them to maintain normal cognition.

Altogether, some of these studies showed greater tolerance to neurodegeneration (atrophy and hypometabolism) in asymptomatic (Aβ-positive) older adults with higher exposure to positive lifestyle factors, which is in line with evidence in AD patients. Other studies, point to brain reserve mechanisms that may help delaying the effects of Aβ deposition on cognition. Studies that directly assess the link between AD neuropathological processes, potential reserve mechanisms and lifestyle are not available yet.

#### Lifestyle Effects on AD Neuroimaging Biomarkers in Cognitively Normal Subjects and APOE ε4 Carriers

**Figure 3:**
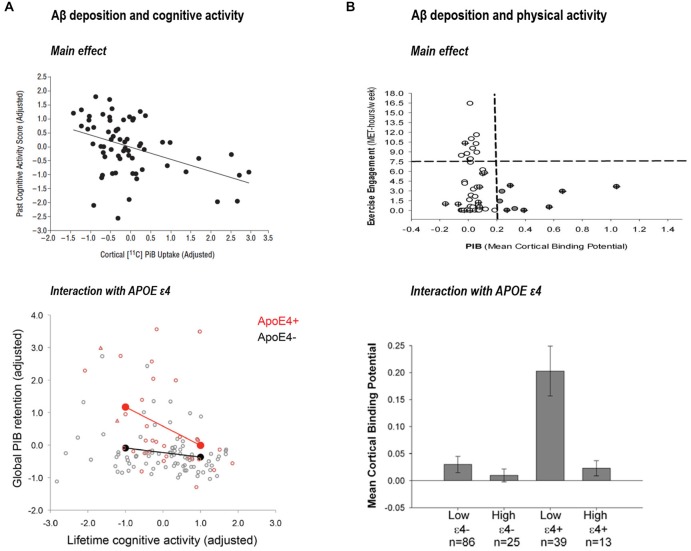
**(A)** Association between past cognitive activity score and cortical PIB-uptake, reprinted from Landau et al. (2012; with permission) and interaction between cognitive activity and APOE ε4 on global PIB retention reprinted from Wirth et al. (2014b; with permission). **(B)** Association between physical activity and PIB mean cortical binding potential reprinted from Liang et al. (2010; with permission) and interaction between physical activity and APOE ε4 on mean cortical binding potential, reprinted from Head et al. (2012; with permission).

Liang et al. (2010) provided novel associations between exercise engagement and biomarkers levels (measured with PET-PIB and CSF levels of Aβ_1–42,_ tau and phospho-tau). They observed that subjects with greater physical activity had lower Aβ deposition. In fact, when they compared exercise scores between individuals with “at risk” biomarkers levels and individuals with “normal” levels those with more altered biomarkers were more sedentary. A later study by Landau et al. (2012) further supported the idea that lifestyle factors may have a direct effect on brain Aβ pathology. They showed an inverse association between Aβ deposition (measured using PIB) and lifelong cognitive activities in cognitively normal subjects. In line with the report in physical activities, healthy older participants in the lowest cognitive activity tertile had Aβ levels similar to patients with AD. This finding was confirmed in a subsequent study from the same laboratory using path analyses and an increased sample size (Wirth et al., 2014a). Further, a series of recent investigations from different laboratories suggest that effects of cognitive and physical activities on Aβ deposition might be exacerbated in those individuals with an APOE ε4 allele (Head et al., 2012; Brown et al., 2013; Wirth et al., 2014b; see Figure 3). Finally, a recent study (Okonkwo et al., 2014) in late middle-age cognitively normal individuals showed that age-related alteration in Aβ deposition, glucose metabolism of the precuneus and hippocampal volume was attenuated in subjects with higher involvement in physical activity.

Similar direct effects on hippocampal atrophy were shown in a longitudinal study by Valenzuela et al. (2008) where complex mental activity across the lifespan was related to reduced hippocampal atrophy in cognitively normal older people. In line with this observation, occupational complexity, specifically supervisory experience at work, has also been related to a lower rate of hippocampal atrophy in cognitively normal older adults (Suo et al., 2012), and higher educational attainment was reported to be related to decreased hippocampal perfusion (Piras et al., 2011). Beyond the hippocampus, an effect of education, occupation and leisure activities on temporal and parietal gray matter volumes and cortical thinning has also been reported (Foubert-Samier et al., 2012; Liu et al., 2012; Arenaza-Urquijo et al., 2013a). However, two studies including a large sample of cognitively normal individuals from ADNI found inconsistent results. A cross-sectional study did not replicate these positive effects of lifestyle factors on AD neuropathological processes (Vemuri et al., 2012). Thus, no significant correlation was found between lifetime or current intellectual activity and Aβ PET, glucose metabolism or hippocampal volumes (however, all these biomarkers were associated with cognitive performance). A subsequent longitudinal study using ADNI subjects (Lo et al., 2013), investigated the effects of cognitive reserve (i.e., education, IQ and occupation) and brain reserve (i.e., intracranial size) proxies on AD pathological progression, including CSF Aβ_1–42_, FDG-PET and hippocampal volumes. Only CSF Aβ_1–42_ was found to be influenced by cognitive reserve proxies in cognitively normal elders.

In summary, emerging evidence suggest that lifestyle factors such as cognitive and physical activity may also have direct effects on Aβ deposition and rates of Aβ accumulation. These direct effects might be restricted to cognitively normal older adults, and may be exacerbated in APOE ε4 carriers. Finally, direct effects of lifestyle in key regions such as the hippocampus and the temporal lobe have also found support in several cross-sectional studies.

## Discussion

**Figure 4:**
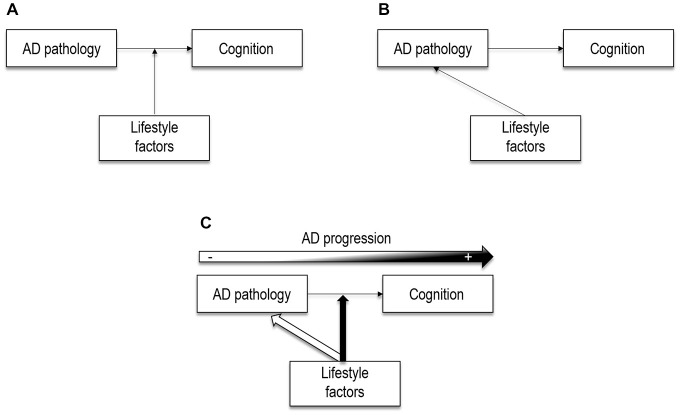
**Theoretical illustrations of the potential effects of lifestyle factors. (A)** Lifestyle factors may act as moderators between brain pathology and cognition, as posited by the cognitive reserve model. **(B)** Lifestyle factors may have a direct (neuroprotective) effect on AD neuropathology, as shown by more recent neuroimaging studies. **(C)** Lifestyle factors may act through both neuroprotective and compensatory pathways, that may coexist. Lifesytle factors may have a direct effect on AD pathological processes in cognitively normal older adults. However, when the pathology reaches a certain level, lifestyle factors may only moderate the relation between AD pathology and cognition. In other words, neuroprotective processess may have a major role in early stages, and compensatory processess later in the disease progression.

Currently, the relationships between lifestyle, AD neuropathology and cognition remain unclear despite the relevance of this question for our understanding ofhealthy aging, and the potential impact for intervention and prevention. From a theoretical perspective, we propose to integrate new evidence in preclinical AD to previous studies in AD in a general model (Figure 4). This model does not aim to capture the full complexity of lifestyle effects on the brain, but attempts to frame our current understanding of the relationships between lifestyle and AD neuropathology over the course of the disease. Neuroimaging studies in AD and cognitively normal elders (including preclinical AD) will be the guiding threat for discussion, while complementary evidence from other fields will be used as supports to discuss on the potential underlying mechanisms.

### Potential Mechanisms Underlying Lifestyle Effects

Lifestyle factors were initially suggested as moderators between brain changes and cognitive performance, under the cognitive reserve hypothesis (Stern, 2002), as represented in Figure 4A. This implies that lifestyle factors may protect against the clinical manifestations of AD rather than influence AD neuropathological processes. Nowadays, growing neuroimaging evidence in cognitively normal individuals points to potential links between lifestyle factors and AD neuropathological expression (Figure 4B), which suggests that the effects of lifestyle might be exerted via both compensatory and neuroprotective mechanisms. In the present review we argue that lifestyle-related compensatory and neuroprotective mechanisms may coexist and play a differential (but not exclusive) role along the disease with neuroprotective mechanisms having a major role in early stages and compensatory mechanisms later in the disease progression (Figure 4C).

#### Compensatory Mechanisms

The abovementioned studies in AD patients, showing more altered biomarkers (hypometabolism, atrophy and Aβ) in patients with higher exposure to positive lifestyle factors, argue in favor of compensatory mechanisms underlying lifestyle effects. According to this view, more pathology is necessary for clinical symptoms to become apparent (Stern, 2009). While, this implies that lifestyle may help delaying or masking cognitive decline, it also implies that once clinical symptoms appear the pathology would be more advanced. This idea is supported by several studies showing that older individuals with higher years of education and/or occupation (Teri et al., 1995; Rasmusson et al., 1996; Unverzagt et al., 1998; Stern et al., 1999; Wilson et al., 2004; Amieva et al., 2005; Hall et al., 2007) and participation in cognitive or leisure activities (Helzner et al., 2007; Hall et al., 2009) had delayed memory decline but a more rapid decline after disease onset. Moreover, this notion is also supported by autopsy studies showing a modifier effect of lifestyle factors in the relation of Aβ pathology to cognition (Del Ser et al., 1999; Bennett et al., 2003; Mortimer et al., 2005) rather than a direct relation between lifestyle factors and development of AD pathology (for a review, see Bennett et al., 2014).

Another important implication of these findings is that lifestyle factors may exert their effect through compensatory mechanisms such as neural compensation. Compensatory mechanisms related to healthy aging and AD have been often reported, and maybe strengthened by lifestyle factors (Park and Reuter-Lorenz, 2009). In fact, neural compensation related to cognitive reserve proxies (i.e., education, IQ and/or leisure activities) has been described in AD and/or MCI patients as compared to older controls using different fMRI task, such as non-verbal memory (Scarmeas et al., 2004), encoding memory (Solé-Padullés et al., 2009) and language (Bosch et al., 2010). Neural compensation for the effects of Aβ and/or hippocampal atrophy on memory have been described in Aβ positive cognitively normal elders (Elman et al., 2014) and MCI patients (Huijbers et al., 2015), however, it is still unclear for which aspects of AD neuropathology patients are able to compensate.

Despite this general agreement for a compensatory perspective in AD, potential direct effects of lifestyle factors (education and/IQ) on AD pathology have also been described in AD patients (Lo et al., 2013; Shpanskaya et al., 2014). Thus, as represented in Figure 4B, an alternative possibility is that lifestyle factors directly impact on AD neuropathological processes.

#### Neuroprotective Mechanisms

Exposure to positive lifestyle factors may protect against the development of the disease for example, by slowing Aβ deposition or rates of hippocampal atrophy (Valenzuela et al., 2008; Liang et al., 2010; Landau et al., 2012). In contrast to previous evidence arguing for compensatory processes in AD patients, evidence for a direct relationship between lifestyle factors and AD neuropathological processes have mainly been reported in cognitively normal older adults. Although only poorly supported by autopsy studies (for a review, see Bennett et al., 2014), this idea has received further support from animal and intervention studies. Thus, researches in animals showed, for example, increased clearance of Aβ and reduced Aβ levels in mice exposed to enriching environments (Lazarov et al., 2005; Costa et al., 2007). Intervention studies on the other hand reported increases in hippocampal size (Erickson et al., 2011) and perfusion (Burdette et al., 2010) with exercise training and biochemical changes in the hippocampus after cognitive training (Valenzuela et al., 2003).

Although this review focuses on brain regions known to be vulnerable to AD, it needs to be noted that these direct/neuroprotective effects of lifestyle factors may extend to other brain areas. In fact, neuroimaging studies in cognitively healthy older adults have shown that lifestyle effects may extend to the frontal lobe, where changes in gray matter and glucose metabolism in cognitively normal elders have been evidenced (Foubert-Samier et al., 2012; Arenaza-Urquijo et al., 2013a). It remains unclear however, whether these changes help maintaining cognitive functioning later in the disease evolution (for example when Aβ deposition becomes elevated). Increase in volume or metabolism in brain areas not sensitive to AD may indeed help coping with the progressing AD pathology (i.e., Aβ deposition and decreased volume and metabolism in AD-sensitive brain areas), which would result in delayed cognitive decline. For example, higher temporal or parietal gray matter volume (Chételat et al., 2010; Johnson et al., 2014) or higher basal metabolism may help coping with the effects of Aβ on cognition (Cohen et al., 2009; Johnson et al., 2014). This more “passive” compensatory mechanism might co-occur with more active mechanisms such as the neural compensation described above. Whether these increases in volume and metabolism are provided by lifestyle experiences or may be related to brain reserve (innate differences) or both, i.e., initial advantages may be promoted by lifestyle, remains unclear and needs to be clarified in longitudinal studies.

#### Compensatory and Neuroprotective Mechanisms Over the Course of Alzheimer’s Disease

It is unlikely that lifestyle factors act exclusively through neuroprotective or through compensatory mechanisms. Indeed, these two mechanisms may coexist. As illustrated in Figure 4C, lifestyle variables may play a double role in preventing AD neuropathology and attenuating the impact of this pathology on cognition via compensatory mechanisms. In fact, despite strong evidence toward compensatory mechanisms in AD patients, there is some neuroimaging evidence for direct protective effects of lifestyle factors in AD, reporting for e.g., slower rates of metabolism decline in high educated AD patients (Lo et al., 2013). Since almost all previous evidence in AD is cross-sectional, it is possible that direct effects on AD neuropathological progression were masked by baseline differences; longitudinal studies might thus allow detecting such direct effects of lifestyle factors on AD pathology.

In the absence of longitudinal studies, cross-sectional studies across different clinical groups (for example healthy older adults, MCI and AD) may offer some important information as regard the differential role that lifestyle factors may play over the course of the disease. For example, differential relationships between lifestyle factors in cognitively normal elders, MCI and AD patients have been reported (Lo et al., 2013), including with measures of cortical thickness (Liu et al., 2012) and whole brain volumes (Solé-Padullés et al., 2009). These studies point to direct effects of lifestyle variables in cognitively normal elders (i.e., increased brain volume, cortical thickness or slower rate of amyloid deposition) and indirect effects (e.g., decreased brain volume or cortical thickness) in patients. These findings converge with those of fMRI studies showing differential patterns of activations in normal older adults vs. patients. Thus, comparing individuals with high vs. low exposure to positive lifestyle variables, lower task-related activation in cognitively normal older adults vs. higher activation in patients have been described. These results might reflect increased neural efficiency in cognitively normal older adults (i.e., less resources are needed to perform a task) vs. neural compensation (i.e., more resources are needed to perform the task) in patients (for a review, see Bartrés-Faz and Arenaza-Urquijo, 2011).

A complementary idea is that increasing Aβ deposition may represent a turning point where lifestyle-related compensatory mechanisms would start taking place. Although compensation for other aspects of aging might exit, within the context of AD this idea looks plausible. Aβ deposition is considered a pivotal event in the AD pathologic cascade that may exacerbate changes in glucose metabolism and atrophy. It is possible that direct effects of lifestyle factors on Aβ deposition only occurs before cognitive impairment. Indeed, this converge with the fact that effects of lifestyle factors on Aβ deposition have been restricted to cognitively normal elders (see above). Thereby, we propose that lifestyle factors may exert their effects mainly through neuroprotective mechanisms in early stages (as evidenced in cognitively normal subjects) and compensatory mechanisms later in the disease progression (as shown in AD patients), as illustrated in Figure 4C. In a simplified scenario where we consider that Aβ-positive subjects are in the pathway toward AD, reports showing that cognitively normal Aβ-positive subjects with higher cognitive reserve proxies had greater atrophy and glucose hypometabolism in AD vulnerable regions (Arenaza-Urquijo et al., 2013b; Ewers et al., 2013) might be interpreted as evidence for a greater tolerance to pathology in these subjects, which allows them to remain cognitively normal while they are more advanced in the pathway to AD. Conversely, cognitively normal Aβ-positive subjects with lower exposure to positive lifestyle variables may have progressed to MCI at this stage of brain alteration (atrophy and hypometabolism). This differential relationship between lifestyle factors and metabolism or gray matter volume in Aβ-positive and Aβ-negative cognitively normal older adults, suggests that the inclusion of older adults at higher risk of developing dementia (for example, presenting high Aβ deposition; Bartrés-Faz and Arenaza-Urquijo, 2011; Arenaza-Urquijo et al., 2013a) or subjects in pre-and post-amyloid plaque stages (Jagust and Mormino, 2011) might be a major confound that needs to be considered in future studies. Furthermore, this might explain some results in previous studies in healthy older adults where this variable was not taken into account, showing associations similar to those found in AD (i.e., negative correlations between positive lifestyle factors and measures of brain integrity) in cognitively normal adults (Coffey et al., 1999; Querbes et al., 2009; Bastin et al., 2012).

It is important to point out that other concepts than cognitive reserve, such as brain maintenance (Nyberg et al., 2012), that emphasize neuroprotective mechanisms related to a higher resistance to pathological processes, may be considered to explain direct effects on AD neuropathological processes. Indeed, a previous review by Barulli and Stern (2013) proposed that cognitive reserve, brain reserve and brain maintenance models may be complementary to explain different aspects of resilience to pathology, including both aspects related to maintaining brain integrity (brain maintenance, Nyberg et al., 2012), and potential compensatory mechanisms (cognitive reserve). In line with this, several authors have discussed this new evidence including the notion that lifestyle variables may have an impact on AD neuropathology (see for example, Vemuri and Mormino, 2013). Here, we emphasize the need to consider the disease progression to improve our understanding of the effects of lifestyle on AD neuropathology.

## Summary and Perspectives

Complementary evidence from AD and preclinical AD researches point to the idea that lifestyle factors such as cognitive, physical and social activities may exert their effects through both neuroprotective and compensatory mechanisms. While studies in AD patients point to lifestyle-related compensatory mechanisms, new evidence in cognitively normal elders with AD neuroimaging biomarkers and preclinical AD subjects suggests that lifestyle factors may directly impact the development of AD neuropathology. In the present review, we propose that including a perspective on disease progression may thus allow a better understanding of lifestyle effects on AD neuropathological processes. Neuroprotective mechanisms may be at work mainly early in the disease progression while compensatory mechanisms may predominate in later stages of the disease. Thus, lifestyle-related neuroprotective mechanisms may no longer be efficient once the pathological processes have progressed. Future research focusing in the relation between lifestyle factors, AD neuropathology, and potential compensatory mechanisms probably using task-related fMRI approaches are needed to confirm this view. Furthermore, studies assessing both lifestyle-related neuroprotective mechanisms and compensatory processes across different clinical and pathological stages of the disease will shed light into this issue.

It needs to be acknowledged that results showing an impact of lifestyle factors on AD neuropathology (for example, on Aβ deposition) in cognitively normal elders are novel and more evidence still needs to accumulate. These results however appear reliable as converging evidence comes from different laboratories (i.e., different samples), and using different lifestyle variables (for example, cognitive activities or physical activities).

As also pointed throughout this review, our understanding of lifestyle effects might be hampered by different issues that need to be considered in future research. First, because of the cross-sectional design of the majority of studies, the effects of lifestyle factors on the rate of decline of AD biomarkers have not been fully assessed. Thus, longitudinal studies are needed to evaluate this question considering potential baseline differences in biomarker alteration. Second, the definition of a valid and accurate quantitative neural measure of cognitive reserve is very challenging. In this regard, functional connectivity measures (reflecting the synchronization of neuronal activity between anatomically separate brain regions, Biswal et al., 1995), may provide new insights into the field. Indeed, functional connectivity might be shaped by experience such as cognitive training (e.g., Jolles et al., 2013; Takeuchi et al., 2013) and thus might be a good measure of experience-dependent brain changes such as that provided by lifestyle. However, evidence relating cognitive reserve measures to functional connectivity remains sparse. In cognitively normal older adults, it has been suggested that education may increase the connectivity between frontal and temporal and parietal regions (Arenaza-Urquijo et al., 2013a). In AD and MCI patients, education may increase the connectivity of the posterior cingulate with other areas of the default mode network (Bozzali et al., 2015). Future studies with a perspective on functional networks will probably provide significant advances to the field. Third, the incomplete understanding of the cascade of events over the course of AD increases the difficulty to interpret lifestyle effects and underlying mechanisms. Probably, a further understanding of the relationships between AD neuroimaging biomarkers will also help us to better understand lifestyle effects. Brain regions (such as temporo-parietal metabolism, or hippocampal volume) that would be affected by AD pathology might be susceptible to lifestyle effects earlier in life, and may then play an important role later in the disease by increasing the threshold of pathology needed to show cognitive impairment. More generally, future studies including both subjects with and without pathology (for example, young subjects) will help understanding lifestyle effects over the life course. In line with this, models including a life course perspective on the effects of lifestyle (for example, Richards and Deary, 2005) may provide a more dynamic perspective to these changes (from childhood to adulthood). Furthermore, comprehensive studies assessing the differential or interactive effects of different lifestyle factors will shed light into the field, since currently the use of different measures across studies blurs the direct comparison between studies.

Overall, lifestyle factors may influence the development and clinical manifestations of AD in several ways. Positive lifestyle variables may continually exert their effects through different mechanisms that in turn help maintaining cognitive performance. Understanding the effects of lifestyle factors across the disease course will help directing therapeutic interventions. If protective effects of lifestyle variables mainly occur in pre-clinical stages, this would imply that lifestyle-based interventions should be initiated in these earliest stages. Complementarily, compensatory mechanisms might be promoted later in the disease which may help decreasing the effects of AD pathology on cognition.

## Author Contributions

EMAU contributed to the conception of the work, drafted and wrote the work. MW and GC contributed to the conception of the work and performed a critical revision for intellectual content. EMAU, MW and GC approved the last version.

## Conflict of Interest Statement

The authors declare that the research was conducted in the absence of any commercial or financial relationships that could be construed as a potential conflict of interest.
